# Single-Shot Readout Performance of Two Heterojunction-Bipolar-Transistor Amplification Circuits at Millikelvin Temperatures

**DOI:** 10.1038/s41598-019-52868-1

**Published:** 2019-11-18

**Authors:** M. J. Curry, M. Rudolph, T. D. England, A. M. Mounce, R. M. Jock, C. Bureau-Oxton, P. Harvey-Collard, P. A. Sharma, J. M. Anderson, D. M. Campbell, J. R. Wendt, D. R. Ward, S. M. Carr, M. P. Lilly, M. S. Carroll

**Affiliations:** 1 Department of Physics and Astronomy, University of New Mexico, Albuquerque, New Mexico 87131 USA; 2 Center for Quantum Information and Control, University of New Mexico, Albuquerque, New Mexico 87131 USA; 3 Sandia National Laboratories, 1515 Eubank Blvd SE, Albuquerque, New Mexico 87123 USA; 4 Département de Physique et Institut Quantique, Université de Sherbrooke, Sherbrooke, Québec J1K 2R1 Canada; 5 Center for Integrated Nanotechnologies, 1515 Eubank Blvd SE, Albuquerque, New Mexico 87123 USA

**Keywords:** Quantum dots, Electronic and spintronic devices, Qubits

## Abstract

High-fidelity single-shot readout of spin qubits requires distinguishing states much faster than the T_1_ time of the spin state. One approach to improving readout fidelity and bandwidth (BW) is cryogenic amplification, where the signal from the qubit is amplified before noise sources are introduced and room-temperature amplifiers can operate at lower gain and higher BW. We compare the performance of two cryogenic amplification circuits: a current-biased heterojunction bipolar transistor circuit (CB-HBT), and an AC-coupled HBT circuit (AC-HBT). Both circuits are mounted on the mixing-chamber stage of a dilution refrigerator and are connected to silicon metal oxide semiconductor (Si-MOS) quantum dot devices on a printed circuit board (PCB). The power dissipated by the CB-HBT ranges from 0.1 to 1 *μ*W whereas the power of the AC-HBT ranges from 1 to 20 *μ*W. Referred to the input, the noise spectral density is low for both circuits, in the 15 to 30 fA/$\sqrt{{\bf{Hz}}}$ range. The charge sensitivity for the CB-HBT and AC-HBT is 330 *μ*e/$\sqrt{{\bf{Hz}}}$ and 400 *μ*e/$\sqrt{{\bf{Hz}}}$, respectively. For the single-shot readout performed, less than 10 *μ*s is required for both circuits to achieve bit error rates below 10^−3^, which is a putative threshold for quantum error correction.

## Introduction

Spin qubits in semiconductors are a promising platform for building quantum computers^1–8^. Significant progress has been achieved in recent years, including demonstrations of extremely long coherence times^9^, high-fidelity state readout^10–13^, high-fidelity single qubits gates^9,14–16^, and two qubit gates^7,16–18^. As the field advances to multiple qubit systems, improvements in single-shot state readout and measurement times will be necessary to achieve fault tolerance. Improving the signal-to-noise ratio (SNR) and bandwidth (BW) of the qubit state detection is critical for both tunnel rate selective readout^2^ and energy selective readout^3^. With the same bit error rate, faster readout will reduce tunnel rate and metastable relaxation or relaxation related errors.

Cryogenic amplification is one way the readout SNR and BW can be improved. Challenges are that: (1) input signals remain relatively small^19–23^ and (2) significant noise and parasitic capacitance is introduced into the measurement circuit when routing the signal out of a dilution refrigerator^24^. Several approaches for cryogenic amplification include: radio-frequency (RF) resonant quantum point contact (QPC) and single electron transistor (SET) circuits^25–32^, gate dispersive RF circuits^33^, Josephson parametric amplification circuits^34^, and cryogenic transistors^35–39^. For single-shot readout, qubit state distinguishability with sensitivity 140 *μ*e/$\sqrt{{\rm{Hz}}}$ has been demonstrated^29^. However, many of these circuits require elements to be mounted at multiple fridge stages and the use of custom on-chip components, adding to the complexity of their implementation. Simpler amplification circuits that use low power transistors mounted directly on the mixing chamber stage with the qubit device thus have significant appeal^38,39^. For example, a proof of principle readout demonstration with a dual stage HEMT achieved T_e_ = 240 mK, gain = 2700 A/A, power = 13 *μ*W, noise referred to input ≤70 fA/$\sqrt{{\rm{Hz}}}$, and 350 *μ*e/$\sqrt{{\rm{Hz}}}$ charge sensitivity^39^.

**Figure 1:**
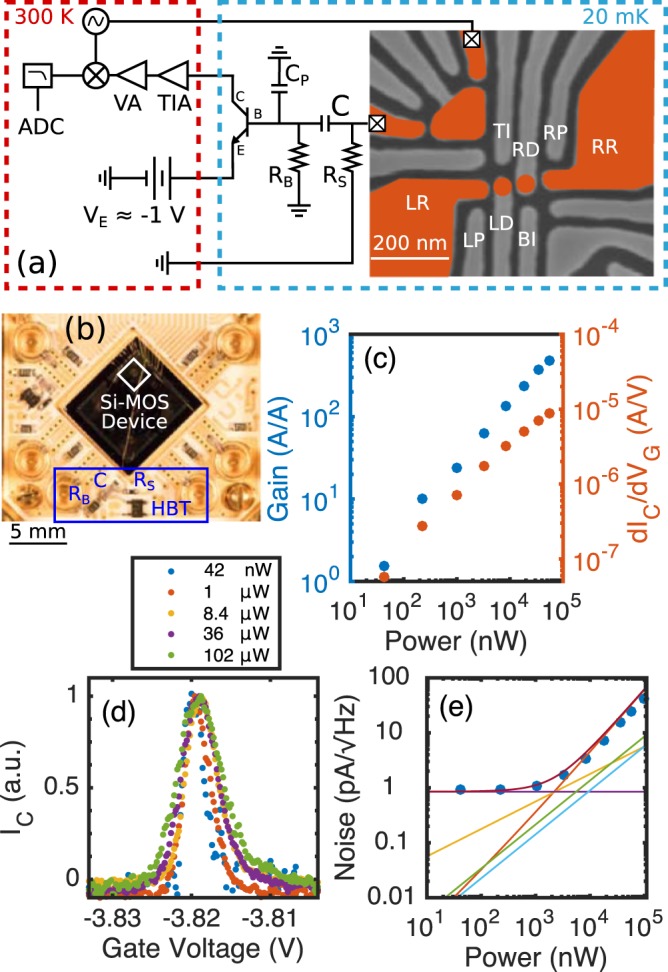
All data in this figure are acquired using about a 74 kHz driving frequency on the SET. (**a**) Schematic diagram of AC-HBT and SEM image of the double quantum dot (DQD) device. Areas of electron accumulation are indicated by false color highlighting of enhancement gates. The charge sensor used to measure the DQD state is in the upper left quadrant, whose source is connected to an AC + DC signal generator, and whose drain is connected to a cryogenic AC-coupled HBT amplification stage. Values of the passive elements are R_B_ = 1 MΩ, R_S_ = 100 kΩ, and C = 10 nF. (**c**) Circuit gain and sensitivity vs. power dissipated by the AC-HBT. (**d**) Normalized CB peak for different AC-HBT gain/power biases. The the blockade region of the CB peak reaches zero current. (**e**) Noise referred to the collector of the AC-HBT for different powers. The measured noise is plotted as blue points. The noise floor of the fridge (purple) (see Section VI in Supplementary Information), shot noise of the base (orange), collector (yellow), SET (light blue), Johnson noise of the shunt resistor (green), and total estimated noise (dark red) are plotted as solid lines.

**Figure 2:**
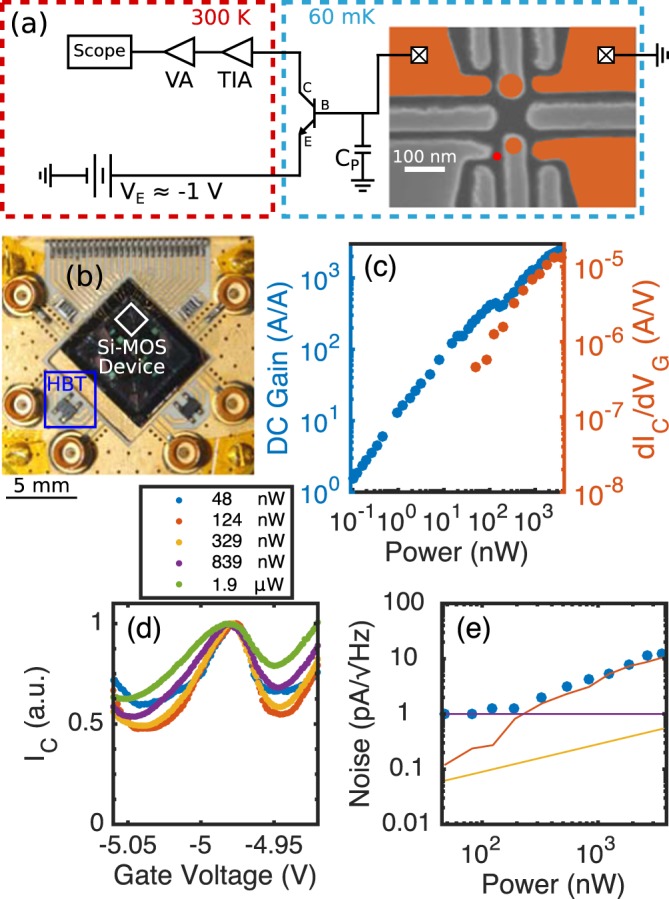
(**a**) Schematic diagram of CB-HBT readout circuit including room temperature amplification and biasing. The SET is represented by the larger, upper orange circle, and the QD is represented by the smaller, lower orange circle. (**b**) Image of the PCB which shows the Si-MOS device and HBT mounted close together. (**c**) DC current gain and sensitivity vs. power dissipated by the CB-HBT. (**d**) Normalized CB peak for different CB-HBT gain/power biases. The blockaded regions of the CB peak do not reach zero current. (**e**) Noise referred to the collector of the CB-HBT at around 7 kHz for different powers. The measured noise is plotted as blue points. For comparison, the noise floor of the fridge (purple curve), base current shot noise (orange curve), and collector current shot noise (yellow curve) are plotted as well.

Silicon-germanium (SiGe) heterojunction bipolar transistors (HBTs) have been demonstrated to operate at liquid helium temperatures^38,40^ as well as millikelvin temperatures in dilution refrigerators^41–44^. The HBT is motivated by low 1/f noise, high R_out_, and possible opportunities to achieve higher gain at the same power relative to HEMTs. Furthermore, there can be bipolar junction transistor (BJT) advantages compared to field effect transistors (FETs) for low input impedance amplifier circuits^45^. Our approach is to use a single SiGe HBT as a cryogenic amplifier at the mixing chamber stage of a dilution refrigerator to improve the SNR and BW of the signal from a SET used as a charge-sensor. We have designed and characterized two different HBT circuits: (1) the AC-coupled HBT circuit (AC-HBT) (Fig. 1(a)) and (2) the current-biased HBT circuit (CB-HBT) (Fig. 2(a)). The CB-HBT simply has the drain of the SET connected to the base of the HBT, while the AC-HBT has the base of the HBT connected to the drain of the SET via a resistor-capacitor (RC) bias tee. Regardless of the coupling between the HBT and SET, the HBT must be DC biased in order to amplify. For either circuit, the silicon metal oxide semiconductor (Si-MOS) device and HBT are mounted on a printed circuit board (PCB) only centimeters apart. The proximity of the HBT amplifier to the SET has the advantages of minimizing parasitic input capacitance and increasing signal before noise from the fridge is added. However, since the mixing chamber stage has a cooling power of around 100 *μ*W at 100 mK, the HBT circuits must operate with powers similar or less in order to avoid heating.

In this letter, we first introduce the two amplification circuits with discussions of gain, sensitivity, bias behavior, and noise. We compare the basic performance and operation of the two amplifiers and extract input-referred noise as well as signal response and heating of the quantum dot electrons. Finally, we compare and discuss the performance for single-shot readout, which somewhat depends on the specific layout of the SET and quantum dot to produce larger signals via increased mutual capacitance.

## AC-HBT Description

The AC-HBT consists of a Si-MOS device that is AC-coupled to an HBT (part number NESG3031M05), which amplifies the SET response to AC source-drain voltage excitation at frequencies higher than around 100 Hz. The SET is integrated into a double quantum dot (QD) device (Fig. 1(a): SEM image), which is made on a Si-MOS platform (see Section I in Supplementary Information).

To operate the AC-HBT, the DC base bias is grounded, and the emitter is biased negatively to support a base-emitter bias *V*_*BE*_ above the cryogenic HBT threshold (about −1.04 V). The bias of the emitter power supply sets the base current, collector current, gain, and dissipated power of the HBT. The HBT current at the collector goes through a high-frequency coaxial line to a room temperature transimpedance amplifier (TIA), then through a voltage amplifier, and finally the signal is demodulated, filtered, and digitized. The TIA is referenced to ground, so the collector-emitter bias equals the base-emitter bias. We find that this configuration optimizes the circuit SNR and also requires only two lines coming from room temperature for the three HBT terminals. Figure 1(c) shows the total AC circuit gain and sensitivity vs. the amount of power dissipated by the HBT. The AC gain is measured by comparing the current of a Coulomb blockade (CB) peak with and without the HBT. The SET current can be measured directly by connecting the output of R_S_ to a room temperature TIA (lowest ground in Fig. 1(a)). The sensitivity of the circuit is defined as the gate-voltage derivative of collector-current (slope) on the side of a CB peak, which is the typical bias point where readout occurs. Sensitivities of 1–5 *μ*A/V are achieved in the operating region of the AC-HBT. Since the AC-HBT is a linear amplifier, the shape of a CB peak remains unaffected by different gain/sensitivity bias points of the AC-HBT (Fig. 1(d)). However, the width of the CB peak broadens at higher biases likely due to heating of the PCB by the HBT. The AC bias across the SET was chosen to be 200 *μ*V_RMS_ in this case to minimize the electron temperature below 200 mK.

Noise spectra are collected for different AC-HBT biases (see Section VI in Supplementary Information), and noise at around 74 kHz is referred to the HBT collector and plotted in Fig. 1(e). The noise displays two different behaviors as power dissipated is increased (Fig. 1(e)). The transconductance of the transistor $(\frac{d{I}_{C}}{d{V}_{BE}})$ increases with power, so it is important to identify where the transistor begins to add appreciable noise. In the low-power limit, the noise dependence is approximately flat at around 1 pA/$\sqrt{{\rm{Hz}}}$, which we attribute to the noise after the HBT dominating any AC-HBT noise. As the AC-HBT power is increased to >1 *μ*W, the noise becomes linearly dependent on power. This behavior is predicted by our estimated shot noise for the base current (Fig. 1(e) orange curve). The estimated total noise is calculated by adding all noise source predictions in quadrature (dark red curve) and aligns well with the total measured noise (blue points).

## CB-HBT Description

The CB-HBT circuit consists of the same commercial HBT model wire bonded from its base terminal directly to the drain of the SET. The SET is integrated into a double QD system consisting of a lithographic QD and a secondary object that has not been definitively identified (i.e., either a QD^46^ or donor^23^). The circuit configuration for the collector and emitter terminals is similar to the AC-HBT, except for the output of the voltage amplifier, which is connected to an oscilloscope with an adjustable sample rate. In Fig. 2(b), the DC current gain and sensitivity are plotted as a function of power. The DC current gain is defined as $\frac{{I}_{C}}{{I}_{B}}$, and the sensitivity is defined as before. The sensitivity of the CB-HBT can reach 5 *μ*A/V between 100–500 nW, whereas the AC-HBT requires >10 *μ*W to reach a similar sensitivity.

The CB-HBT acts as a current bias, so there is always current through the SET (see Section II in Supplementary Information). In regions of Coulomb blockade, the HBT base-emitter voltage will shift on the order of the charging energy of the SET in order to maintain a relatively constant current through the circuit. To show the current-biasing effect, a CB peak is plotted for different CB-HBT gain values in Fig. 2(d), and the current is normalized to the value at the top of the CB peak. Although the current in the blockaded regions of the CB peak is much different from a voltage-biased configuration, the slope of the sides of the CB peak appear to be less affected by the current-biasing (sensitivities of 1–5 *μ*A/V are achieved for either circuit). We note that the effect of current bias on Coulomb blockade is independent of the HBT presence (Fig. S3 in Supplementary Information).

The noise referred to the collector of the CB-HBT is examined at around 7 kHz (Fig. 2(e)). Similar qualitatively to the AC-HBT, the lower power region is dominated by noise after the HBT around 1 pA/$\sqrt{{\rm{Hz}}}$ (purple curve). As power is increased, the measured noise (blue points) begins to increase, which follows the estimated behavior of the base current shot-noise (orange curve) (see Section VI in Supplementary Information).

## Amplifier Performance Comparison

**Figure 3:**
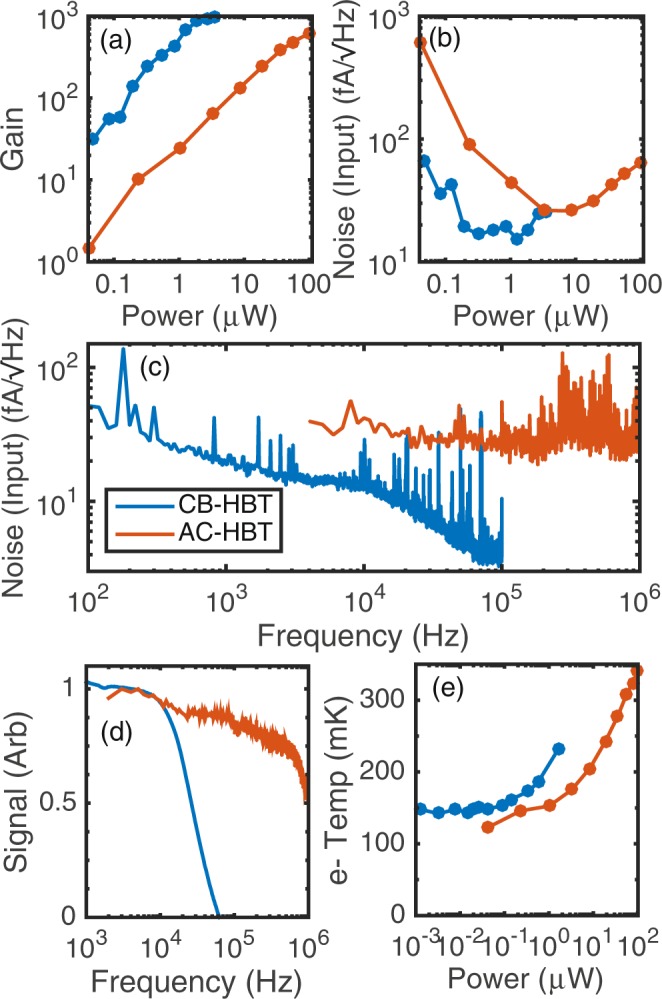
Blue coloring represents CB-HBT data, and orange coloring represents AC-HBT data. (**a**) Gain of both circuits as a function of power. Calculated gain of the CB-HBT is shown (Section V in Supplementary Information). (**b**) Minimum input-referred noise as a function of power. CB-HBT has minimum of 19 fA/$\sqrt{{\rm{Hz}}}$ at 800 nW, and AC-HBT has minimum of 26 fA/$\sqrt{{\rm{Hz}}}$ at 8.4 *μ*W. (**c**) Input-referred noise spectrum of both circuits for power that minimizes noise. The origin of the noise fluctuations at higher frequencies is unclear at this time. (**d**) Signal response (in normalized arbitrary units) for both circuits as a function of frequency. The CB-HBT has a -3 dB point at around 20 kHz, and the AC-HBT has a -3 dB point at around 650 kHz. (**e**) Electron temperature vs. power for both circuits. Base electron temperatures are between 120–150 mK.

We next compare the performance of both amplifiers with respect to power dissipated. The first metric examined is gain as power is increased. The gain of the AC-HBT is simply the measured gain of the amplifier, however the gain of the CB-HBT circuit is not as simple to extract. The small-signal resistance of the SET (r_set_) must be known in order to calculate the CB-HBT gain (see Section V in Supplementary Information). Since the SET is directly connected to the HBT, we cannot measure r_set_. Instead, we use the value of r_set_ (3 MΩ) which best follows the measured noise behavior in Fig. 2(e) to estimate the gain. We plot this estimated gain of the CB-HBT circuit and compare to the measured gain of the AC-HBT circuit in Fig. 3(a). We observe that the CB-HBT circuit achieves higher gain at lower powers, including operating with gain over 400 at a power around 1 *μ*W.

We next compare the noise referred to the input of the HBT for each circuit. Noise is referred to the input using the gain values in Fig. 3(a). We measure the noise spectrum for each circuit at different bias points and choose the frequency which minimizes the noise. The frequency chosen for the AC-HBT circuit was around 74 kHz, and the frequency for the CB-HBT circuit was around 7 kHz. When the input-referred noise is plotted as a function of power (Fig. 3(b)), we observe a minimum noise operating point for either circuit. At low powers, the noise is likely dominated by triboelectric noise due to the fridge and input noise of the room temperature TIA. At higher powers, the HBT amplifiers begin injecting appreciable noise into the circuit, therefore the overall noise increases. The CB-HBT circuit achieves a minimum noise of 19 fA/$\sqrt{{\rm{Hz}}}$ at a power around 800 nW, while the AC-HBT circuit achieves a minimum noise of 26 fA/$\sqrt{{\rm{Hz}}}$ at a power around 8.4 *μ*W.

For the powers that minimize noise for each circuit, we plot the input-referred noise spectrum for both circuits as a function of frequency (Fig. 3(c)). The noise spectrum of the CB-HBT is plotted out to 100 kHz, since its bandwidth is less than 100 kHz. The 1/f-like behavior of the noise at lower frequencies is assumed to be due to charge noise in the Si-MOS device. In the overlapping region around 10 kHz, the noise for the CB-HBT is significantly lower than the noise for the AC-HBT.

Figure 3(d) shows the frequency dependence of an input signal for both amplification circuits up to 1 MHz. The AC-HBT has a -3 dB point at around 650 kHz, and the CB-HBT has a -3 dB point at around 20 kHz, which implies significantly lower BW than the AC-HBT. The origin of this lower BW is not well understood. Using pessimistic numbers, the frequency pole of the SET resistance (assuming 1 MΩ) and the parasitic capacitance between the SET and the base junction (assuming 1 pF) should only limit the -3 dB point to around 160 kHz. In addition, 4 K simulations of this circuit also yielded around 160 kHz -3 dB BW^47^ . Improvements and understanding of the BW of the CB-HBT will be important in future work.

Heating of electrons in the QD due to the operation of the connected HBT is a concern, therefore we examined the dependence of electron temperature on HBT amplifier bias (Fig. 3(e)). For the CB-HBT, we find that the minimum electron temperature observed is around 150 mK. Heating of the QD begins where the CB-HBT is operating with over 100 gain at 100 nW, therefore the CB-HBT circuit can amplify well with an electron temperature around 160–200 mK. For the AC-HBT, the minimum electron temperature was around 120 mK. When the AC-HBT bias is increased up to 3.24 *μ*W, the electron temperature remains near the minimum temperature. For powers above this threshold, the electron temperature increases approximately linearly with power. Nonetheless, an electron temperature of 200 mK is used for the bias condition that provides the minimum amplifier noise.

## Single-Shot Results Comparison

**Figure 4:**
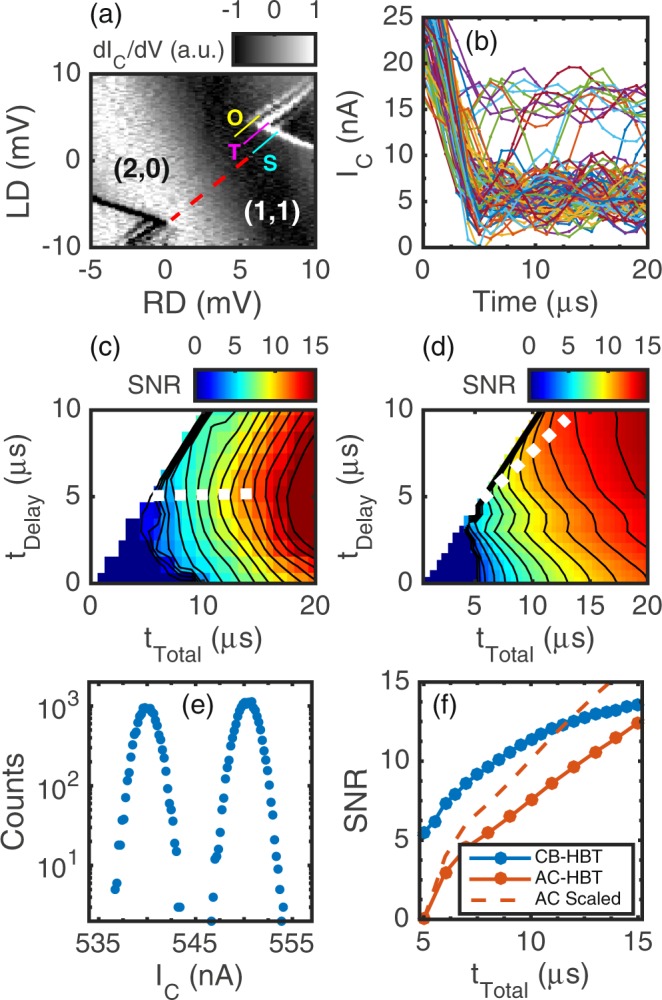
(**a**) Measurement pulse signal (derivative) rastered about the (2,0)–(1,1) anti-crossing for the AC-HBT device. Three distinct latched lines are present. (**b**) 100 single-shot traces of the readout portion of the pulse for the AC-HBT device. Signal separation begins to occur around 4 *μ*s. (**c**) 2D SNR plot for the CB-HBT readout. (**d**) 2D SNR plot for the AC-HBT readout. (**e**) Example histogram from the CB-HBT readout. (**f**) SNR vs. minimum total measurement time for both circuits, which corresponds to the white dashed line in (**c**,**d**). The greater gain of the CB-HBT compensates for the lower bandwidth relative to the AC-HBT. The AC-HBT is also shown scaled by 34% to compare more directly to the CB-HBT, which had a larger SET signal.

We compare both HBT amplifiers by performing single-shot readout of latched charge states^10^. Both Si-MOS quantum dot devices are tuned to the few electron regime and the spin filling of the last few transition lines are verified with magnetospectroscopy. Figure 4(a) shows the result of a three-level pulse sequence in the AC-HBT device where: 1) the system is initialized into (1,0), 2) ground and excited states are loaded in (2,0), and finally 3) the measurement point (signal plotted) is rastered about the (2,0)–(1,1) anti-crossing. When measuring for 30 *μ*s, three latched lines are present, which indicates spin blockade for an excited state triplet (T), a second excited state triplet (O), and a lifting of the spin blockade for the ground state singlet (S). We assign T as a valley triplet with valley splitting of 140 *μ*eV and the O as an orbital triplet with orbital splitting of 280 *μ*eV. For all single-shot measurements, we remove the state O from the available state space by energy selective loading of the (2,0) state.

For both circuits, a mixture of (2,1) and (2,0) charge states are read out in the reverse latching window. Figure 4(b) shows 100 individual single-shot traces of the readout portion of the pulse for the AC-HBT device. Significant feedthrough is observed in the first few *μ*s of the readout pulse, likely due to attenuators connecting the conductor of the high BW lines to the ground of other lines including the emitter bias line. State distinguishability does not begin to occur until about 4 *μ*s, and then the pulse relaxes to two distinct states after about 7 *μ*s. Extracting the SNR from these traces is done by waiting a certain amount of time, t_delay_, and then averaging the signal for a certain amount of time, t_integration_. Histograms of the delayed and averaged shots are compiled and fit to a double Gaussian distribution (Fig. 4(c)). The signal is defined as the separation of the center of the Gaussian peaks, and the noise is defined as the average of the standard deviations of the Gaussian peaks. The SNR is defined as the signal divided by the noise.

The extracted SNR for a given delay and total time (t_delay_ + t_integration_) is plotted in Fig. 4(d,e). Contours are drawn for each SNR integer on both plots, where the leftmost part of a contour line reveals the minimum total measurement time required to reach a given SNR. We plot the SNR vs. minimum total measurement time in Fig. 4(f) for both circuits. The CB-HBT reaches greater SNR at any given time in the 15 *μ*s plot range. Both circuits achieve SNR > 7 in t_total_ < 10 *μ*s, which corresponds to a bit error rate < 10^−3^ and marks a significant improvement over the equivalent t_total_ = 65 *μ*s in previous work^10^. In particular, the CB-HBT is able to reach SNR > 7 in t_total_ ≈6 *μ*s, which represents over a factor of ten improvement from the previous work^10^. The charge sensitivity for the CB-HBT is 330 *μ*e/$\sqrt{{\rm{Hz}}}$ (*τ*_int_ = 6 *μ*s, SNR = 7.4), and the charge sensitivity for the AC-HBT is 400 *μ*e/$\sqrt{{\rm{Hz}}}$ (*τ*_int_ = 9 *μ*s, SNR = 7.5). We note that the SET in the CB-HBT device had around 34% more signal due to larger mutual capacitance (Section I in Supplementary Information) which may contribute to the larger SNRs.

## Conclusion

We compare the performance of two cryogenic amplification circuits: the CB-HBT and the AC-HBT. The power dissipated by the CB-HBT ranges from 0.1 to 1 *μ*W, whereas the power of the AC-HBT ranges from 1 to 20 *μ*W. Referred to the input, the noise spectral density is low for both circuits in the 15 to 30 fA/$\sqrt{{\rm{Hz}}}$ range. The charge sensitivity for the CB-HBT and AC-HBT is 330 *μ*e/$\sqrt{{\rm{Hz}}}$ and 400 *μ*e/$\sqrt{{\rm{Hz}}}$, respectively. For single-shot readout performed, both circuits achieve SNR > 7 and bit error rate < 10^−3^ in times less than 10 *μ*s.

The AC-HBT requires more relative overhead for implementation than the CB-HBT. The AC-HBT includes three additional surface mounted passive elements (Fig. 1(a)), which can be optimized to produce better SNR. Additionally, the AC-HBT has a two-dimensional bias space via the base bias and emitter bias, whereas the CB-HBT is only biased via the emitter bias. However, the AC-HBT is a linear gain circuit and can be used with discrete HEMTs^48^ and HBTs, providing more opportunity to optimize the transistor. Ideally, the transistors would have greater transconductance (g_m_) and a more ideal dependence on I_C_ than the HBTs used in this work (see Section IV in Supplementary Information). In the present demonstration of the AC-HBT, heating of electrons occurred at powers which minimized noise. Introducing a second AC-HBT stage is relatively straightforward and may allow the first stage to run at powers which don’t heat the electrons and minimize the noise further. In addition, the second stage could be mounted further away from the Si-MOS PCB and reduce local heating.

## Supplementary information

Supplementary Information

## Data Availability

Data available upon request.
