# Supplementary material for: Single-Shot Readout Performance of Two Heterojunction-Bipolar-Transistor Amplification Circuits at Millikelvin Temperatures
Source: Sci Rep. 2019 Nov 18;9:16976. doi: 10.1038/s41598-019-52868-1 (PMC6861249; doi:10.1038/s41598-019-52868-1)
Supplement: Supplementary file 1 — Supplementary Information [file 41598_2019_52868_MOESM1_ESM.pdf]

Supplementary Information for Single-Shot Readout Performance of Two Heterojunction-Bipolar-Transistor Amplification Circuits at Millikelvin Temperatures

M. J. Curry,[1,2,3,a)] M. Rudolph,[3,a)] T. D. England,[3] A. M. Mounce,[3] R. M. Jock,[3] C. Bureau-Oxton,[4,3] P. Harvey-Collard,[4,3] P. A. Sharma,[3] J. M. Anderson,[3] D. M. Campbell,[3] J. R. Wendt,[3] D. R. Ward,[3] S. M. Carr,[3] M. P. Lilly,[3,5] and M. S. Carroll[3]

[1)] *Department of Physics and Astronomy, University of New Mexico, Albuquerque, New Mexico, 87131, USA*

[2)] *Center for Quantum Information and Control, University of New Mexico, Albuquerque, New Mexico, 87131, USA*

[3)] *Sandia National Laboratories, 1515 Eubank Blvd SE, Albuquerque, New Mexico, 87123, USA*

[4)] *Département de physique et Institut quantique, Université de Sherbrooke, Sherbrooke (Québec) J1K 2R1, Canada*

[5)] *Center for Integrated Nanotechnologies, 1515 Eubank Blvd SE, Albuquerque, New Mexico, 87123, USA*

I. SET GEOMETRIES AND DETAILS

The SET connected to the AC-HBT uses a single layer doped poly-Si electrode structure on 50 nm thick SiO_2, providing a mobility of 19,500 cm^2/Vs at 4 K. The poly-Si gate layer is etch-defined into electrodes that control the formation of the SET (upper left in Figure 1(a) SEM image) and two quantum dots (under gates RD and LD). Regions of electron enhancement are indicated by the highlighted regions.

The Si-MOS device in the CB-HBT circuit is similar to the Si-MOS device in the AC-HBT circuit with the exception that the SiO_2 layer is 35 nm thick and the bottom layer is isotopically purified silicon (500 ppm ^{29}Si). The ^{28}Si isotope has no net nuclear spin, therefore it is ideal for qubits to be formed in because decoherence due to magnetic noise is highly suppressed. Phosphorous (^{31}P) donor atoms are imbedded in the ^{28}Si layer using ion implantation near where the quantum dot is intended to be formed (red dot in Figure 2(a) SEM image).

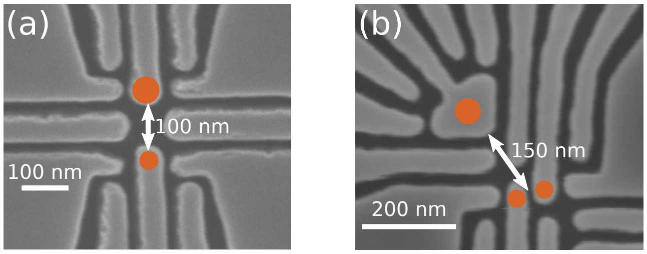

Figure S1. (a) SEM image of Si-MOS device used in CB-HBT circuit. The edge of the SET (larger orange dot) is roughly 100 nm away from the quantum dot (smaller orange dot). (b) SEM image of the Si-MOS device used in the AC-HBT circuit. The proximity of the SET to the double quantum dot system is 50% further away at roughly 150 nm.

The CB-HBT and AC-HBT were characterized using different Si-MOS devices possessing different electrostatic gate layouts (Figure S1). The geometry of the gate layout affects the mutual capacitance between the SET and the quantum dot. More capacitive coupling results in larger changes in the electrochemical potential of the charge-sensor for a given quantum dot charging event[1]. Since changes in electrochemical potential of the charge-sensor result in changes in current through the charge-sensor, larger changes result in larger signal. Therefore, more mutual capacitance leads to larger readout signals, faster readout times, and higher readout fidelity.

The gate geometry used in the Si-MOS device connected to the CB-HBT had the SET 33% closer to the quantum dot than in the Si-MOS device connected to the AC-HBT. The closer SET proximity in the CB-HBT resulted in an increase in sensitivity of approximately 34%. We compare the sensitivity of both circuits by dividing the voltage shift of the dot occupancy transition by the charge-sensor Coulomb blockade peak period. For the CB-HBT, the voltage shift was 18 mV and the charge-sensor period was 337 mV (5.34% change). For the AC-HBT, the voltage shift was 12 mV and the charge-sensor period was 350 mV (4% change). Therefore, the SET in the CB-HBT was around 34% more sensitive to charging events than the AC-HBT.

II. CURRENT-BIASING EFFECT OF CB-HBT CIRCUIT

Since the node that connects the SET source to the HBT base is floating, the bias across the SET cannot be set to a fixed voltage in the CB-HBT circuit. Verilog-A models were created to simulate the behavior of the circuit when biasing the SET through multiple regions of Coulomb blockade via an electrostatic gate. As the SET resistance changes due to Coulomb blockade, the source-drain bias across the SET changes to allow current to

---

[a)] M. J. Curry and M. Rudolph contributed equally to this work.

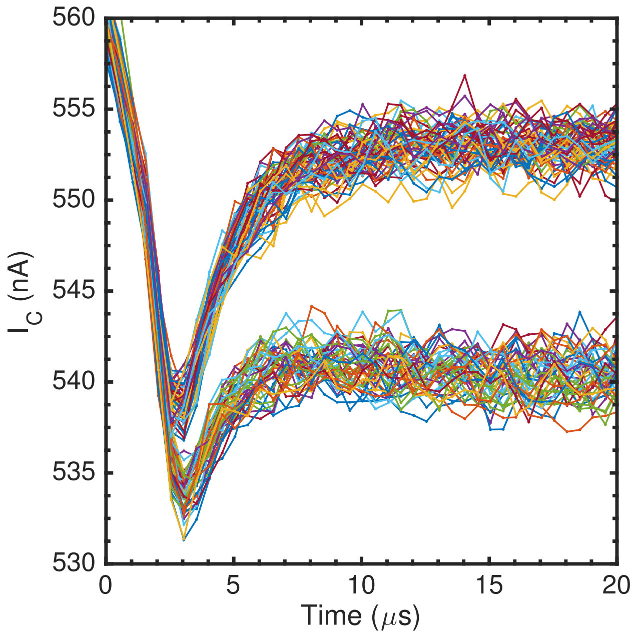

Figure S2. 100 single-shot traces for the CB-HBT charge readout. Slower response time is compensated for by larger signal separation at earlier times relative to the AC-HBT readout.

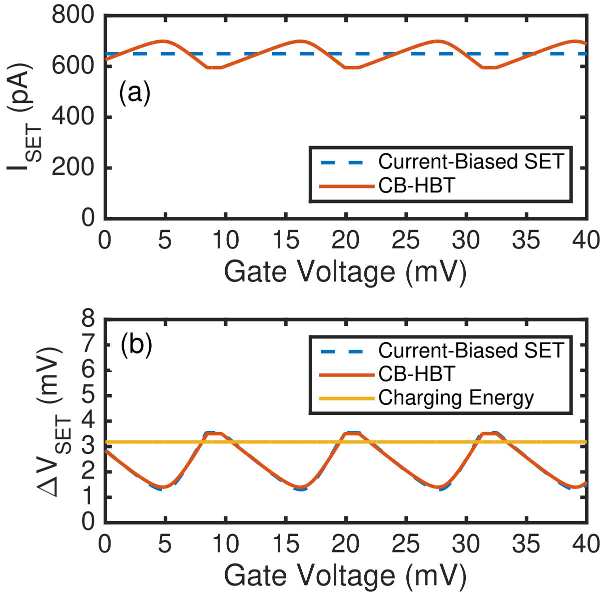

Figure S3. Comparison of CB-HBT and current-biased SET Verilog-A models. The top plot shows the drain current of the SET as a function of gate voltage. The current is modulated much less in this condition than in a constant voltage-biased circuit. The bottom plot shows voltage across the SET as a function of gate voltage. The overlap between the two curves shows that the CB-HBT circuit is effectively equivalent to current-biasing the SET.

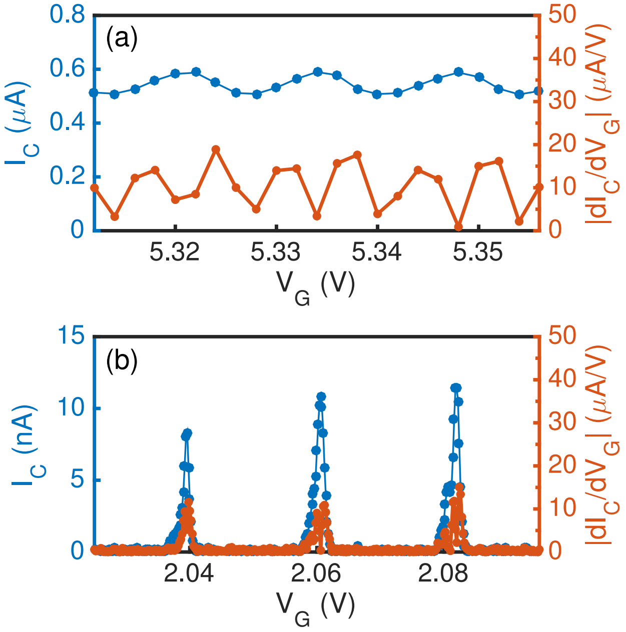

Figure S4. (a) Coulomb blockade peaks for the CB-HBT (blue). The absolute value of the sensitivity is plotted as orange points. Since there is almost always positive or negative blockade slope, the absolute value of the sensitivity remains positive for most of the range plotted. (b) Coulomb blockade peaks for the AC-HBT (blue). The absolute value of the sensitivity is plotted as orange points as well.

flow into the base of the HBT (Figure S3(b)). In order for this to happen, the HBT trades base-emitter voltage for minimal impact to operation. Although the trade in voltage results in a relatively small change in HBT collector current during, for example, a single-shot readout event, this signal is approximately 100 larger than the SET source-drain signal without an HBT (e.g. $\Delta I_C = 10$ nA vs. $\Delta I_{SET} = 100$ pA).

The Verilog-A model estimates the small signal resistances as: $r_{set} = 200$ kΩ and $r_\pi = 10$ MΩ (where r_π is the small signal resistance of the base-emitter junction). Most of the emitter bias voltage is across the base-emitter junction at all times (since $r_{set} << r_\pi$), therefore the CB-HBT is a current-biasing circuit. The current-biasing behavior is highlighted in Figure S4(a), where three Coulomb blockade peaks are plotted. For comparison, three Coulomb blockade peaks are plotted for the AC-HBT case (Figure S4(b)). The CB-HBT amplified peaks are broadened by the current-biasing effect and the blockade region never reaches zero current as it would with a smaller constant voltage bias. The AC-HBT amplified peaks are much narrower and minimally broaden due to having a constant, small voltage bias regardless of HBT power. Comparable sensitivities can be achieved for either circuit around 10 μA/V.

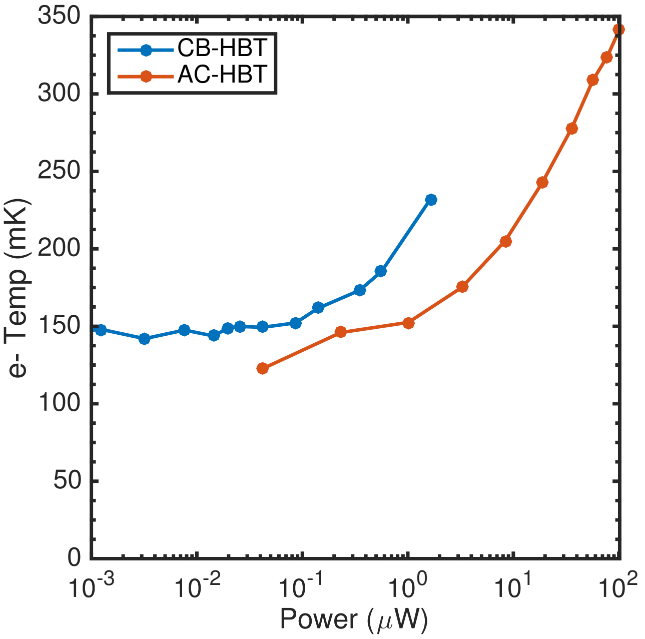

Figure S5. Electron temperature vs. power for both circuits. Base temperatures are between 120–150 mK. Both circuits operate in the 160–200 mK range for single-shot data taken. All temperature measurements have an uncertainty of ±5 mK.

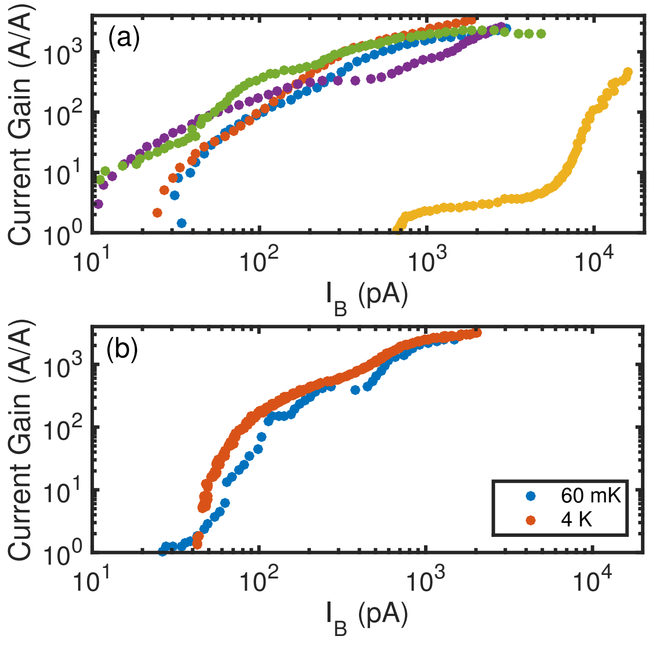

Figure S6. (a) Example plots of current gain vs. base current for different HBTs. Several curves reach current gain > 1000 for base currents < 500 pA. The HBT corresponding to the yellow curve is subpar since it requires base current > 10 nA to reach current gain > 1000. (b) Current gain vs. base current at different temperatures for HBT used in the CB-HBT circuit. There is a slight difference in the two curves, however the performance at 60 mK is enough to efficiently amplify and perform single-shot readout.

III. ELECTRON TEMPERATURE MEASUREMENT

Heating of electrons in the quantum dot due to the operation of the connected HBT is a concern, therefore we examined the dependence of electron temperature on HBT amplifier bias (Figure 3(e)). For the CB-HBT, the electron temperature of the QD was measured by extracting the width of a Coulomb blockade peak of the QD (not SET) as a function of fridge temperature. The QD was tuned to a transport regime where the QD was approximately equally tunnel-coupled to both reservoirs and there were around 10 electrons in the QD. The source-drain bias was reduced to 5 μV_{rms} to avoid bias heating. A Coulomb peak was chosen where a minimum width was observed in Coulomb diamond measurements. After extracting the lever-arm of the gate used to measure the broadening (13 μeV/mV), we find that the minimum linewidth yields an electron temperature around 150 mK. Heating of the QD begins where the CB-HBT is operating with over 100 gain, therefore the CB-HBT circuit can amplify well while heating the electrons to 160–200 mK.

For the AC-HBT setup, the base electron temperature was around 120 mK. This is confirmed by the measurements of the electron temperature when measuring the SET signal directly through the shunt resistor (R_S in Figure 1(a)) with the HBT turned off. With the HBT on, The electron temperature is deduced by measuring the Fermi-Dirac linewidth of the (1,0)-(2,0) charge transition. When the AC-HBT bias is increased up to 3.24 μW, the electron temperature remains near the base temperature (Figure S5). For powers above this threshold, the electron temperature increases approximately linearly with power. This might be due to local heating of the PCB and wires, which increase the temperature of the nearby device[2]. No effort has been made to heat sink the AC-HBT in this experiment, so further tests with various heat sinking options will be performed to minimize the increase in electron temperature. Nonetheless, an electron temperature of 200 mK is achieved for the bias condition that provides the minimum amplifier noise.

IV. HBT CHARACTERIZATION

Before being used in either amplification circuit, HBTs are initially characterized in liquid helium at 4 K using PCBs with eight HBTs mounted on them. We find that HBT performance at 4 K—particularly current gain vs. base current—changes minimally when HBTs are cooled down to 20–60 mK in a dilution refrigerator (Figure S6(b)). This is most likely due to the charge-carrier transport mechanism changing from a drift-diffusion regime (temperature dependent) to a tunneling regime (barrier dependent) at around 30 K[3].

In order to characterize HBTs, Keithley 2400 source-measure units are used as current meters and connected

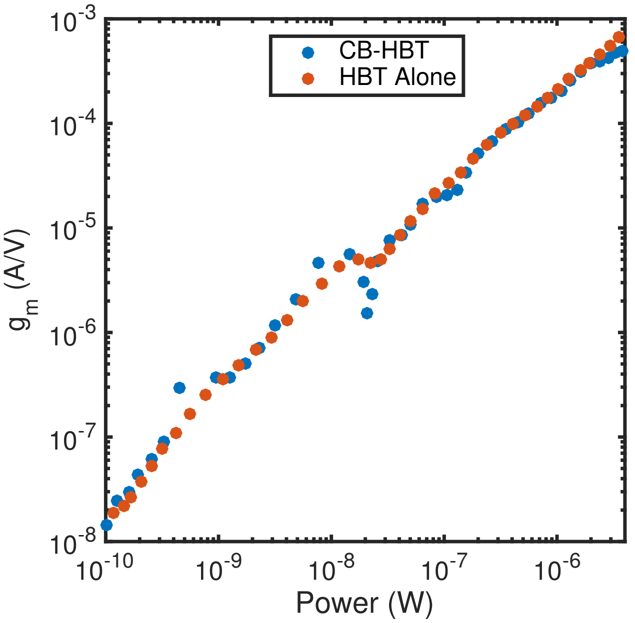

Figure S7. Transconductance vs. power for the CB-HBT (SET connected to HBT) and the same HBT without an SET connected. The data overlaps for both cases, therefore the transconductance can be reliably measured directly in the CB-HBT (assuming $r_{set} \ll r_\pi$).

to the HBT base and collector terminals. A power supply (emitter bias) is connected to the HBT emitter terminal and used to bias the HBT to different operating regimes. The emitter bias has to reach approximately -1 V for the HBT to begin operating in an amplifying regime. As the emitter bias is changed from -1.00 V to around -1.07 V, the collector and base current begin to increase exponentially. The current gain, defined by dividing the collector current by the base current, also increases exponentially as emitter bias changes.

Previous measurements without HBT amplification circuits indicate that the SET current should be below several hundred pA in order to avoid QD electron heating. For the CB-HBT, we select HBTs based on their current gain at low base currents. Around 20% of HBTs characterized will have current gain $>$ 100 at base current $<$ 200 pA (Figure S6(a)). For the AC-HBT, the transconductance (g_m) is the only metric required for selection. Since the HBTs were fabricated with g_m as a primary metric, $>$ 80% of HBTs are usable for the AC-HBT circuit even at low temperatures. However, g_m does not scale ideally in these HBTs at cryogenic temperatures. For a given HBT, $g_m \propto I_C^n$, where $n = 1$ in normal conditions. In the HBTs used in this work, $n \approx 0.8$, which leads to suboptimal SNR at higher power.

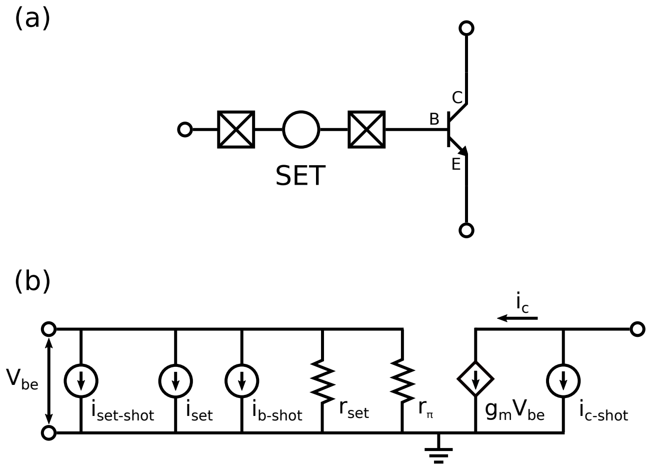

Figure S8. (a) CB-HBT circuit schematic for reference. (b) CB-HBT effective circuit model. The shot noise current source, $i_{b\text{-shot}}$, is in parallel with r_{set} and r_π. Most of the shot noise does not enter the base of the HBT because $r_{set} << r_\pi$. The signal, i_{set}, is also shown, which is amplified according to Equation S1.

V. CB-HBT SMALL SIGNAL GAIN

The gain of the CB-HBT is calculated using a standard BJT small-signal model. A small voltage fluctuation at the base node is usually converted to a large current fluctuation at the collector node by the transconductance, $g_m = \frac{di_c}{dv_{be}}$. This voltage fluctuation is usually the small-signal base-emitter junction resistance, r_π, multiplied by the base current. However, in the case of the CB-HBT, $r_{set} \,||\, r_\pi$, therefore the parallel combination of the two resistances is required to calculate gain:

$$\text{gain}_{CB} = \frac{i_c}{i_{set}} = g_m(r_{set} \,||\, r_\pi) \tag{S1}$$

VI. NOISE MODELS

Sources of noise in the HBT amplification circuits include: shot noise, Johnson noise, triboelectric noise associated with the coaxial lines coupled to fridge vibration[4], room temperature amplifier noise, and other instrumental noise. At relatively low power operation regimes ($<$ 1 μW for the AC-HBT and $<$ 200 nW for the CB-HBT), the noise due to vibrations in the fridge dominates at around 1 pA/$\sqrt{\text{Hz}}$. The input noise spectral density of the room temperature amplifier is relatively low (100–500 fA/$\sqrt{\text{Hz}}$), therefore we focus on noise sources much more dominant. When either circuit is operating in a regime appropriate for single-shot readout, the base shot noise is greater than the collector shot noise (Figures 1(e) and 2(e)). For the SET shot noise in either case, we do not consider a Fano factor, which would reduce the noise for a given power[5,6]. The total noise for either circuit

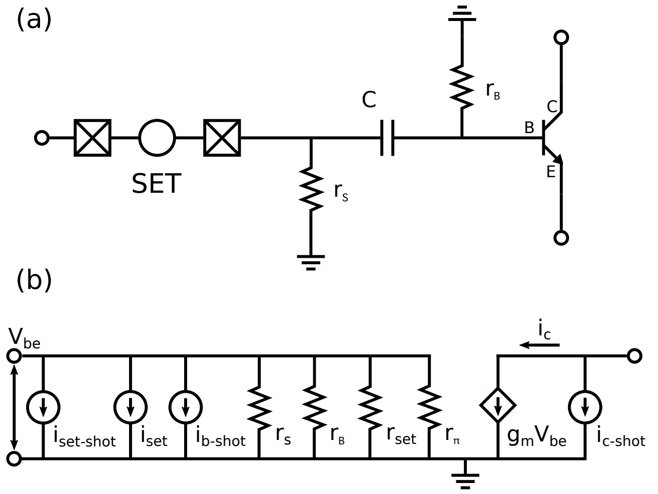

Figure S9. (a) AC-HBT circuit schematic for reference. (b) AC-HBT effective circuit model with signal, $\mathrm{i_{set}}$, also shown. The model is similar to the CB-HBT with two new resistors added in parallel, $\mathrm{r_S}$ and $\mathrm{r_B}$.

is calculated by assuming noise sources are independent processes and adding noise sources in quadrature.

Noise modeling for the CB-HBT circuit is nontrivial because of current division at the HBT base node since $r_{set} \ll r_{\pi}$. The SET and base current are reduced to a Norton equivalent circuit, and the HBT is reduced to $\mathrm{r_\pi}$ connected to a current source which takes voltage fluctuations ($\mathrm{v_{be}}$) across $\mathrm{r_\pi}$ and converts them to collector current via the transconductance, $\mathrm{g_m}$. For the CB-HBT, the noise model is a shot noise current source ($i_{b-shot} = \sqrt{2\,e\,I_B\,\Delta f}$, where I_B is the DC base current, and Δf is the bandwidth centered on frequency f) in parallel with $\mathrm{r_{set}}$ and $\mathrm{r_\pi}$ (Figure S8(b)). Since $r_{set} \ll r_{\pi}$, most of the shot noise current goes through the SET to ground, and a much smaller amount enters the HBT base and is amplified. The amplified base shot noise is shown in Equation S2:

$$i_{\text{b-shot-amp}} = i_{\text{b-shot}}\,\text{gain}_{CB} = i_{\text{b-shot}}\,g_m\,(r_{set}\,||\,r_{\pi}) \quad \text{(S2)}$$

This amplified base shot noise is estimated in Figure 2(e) as the orange curve where $\mathrm{g_m}$ and $\mathrm{r_\pi}$ are calculated from Gummel plots of the HBT and $\mathrm{r_{set}}$ is assumed to be 3 MΩ, which was verified in later measurements with the HBT disconnected from the Si-MOS device.

The noise model for the AC-HBT is similar to the CB-HBT with $\mathrm{r_S}$ and $\mathrm{r_B}$ added in parallel to $\mathrm{r_{set}}$ and $\mathrm{r_\pi}$. The coupling capacitor, C, is considered a short at the frequencies appropriate to model noise in the AC-HBT. The Johnson noise of $\mathrm{R_S}$ in the AC-HBT circuit is $v_{s-jn} = \sqrt{4\,k_B\,T\,R_S\,\Delta f}$ (where T is the temperature) and does not contribute significantly in the single-shot operation regime. Since the AC-HBT has a separate current to bias the base-emitter junction, $I_{SET} \neq I_B$, therefore the base shot noise and SET shot noise are considered separately. However, $I_{SET} < I_B$, so the base shot noise is always dominant in amplifying regimes.

VII. AC-HBT BIAS TEE PARAMETERS

The bias tee parameters for the AC-HBT were chosen to be $\mathrm{R_S}$ = 100 kΩ and C = 10 nF, which sets a high pass filter at 160 Hz. Operating the circuit at frequencies higher than 160 Hz aids in avoiding higher noise levels at lower frequency due to 1/f-like noise behavior in the system.

The shunt resistance value is chosen to be less than $\mathrm{r_{set}}$ (100s of kΩ) so that most of the SET bias voltage drops across the SET.

REFERENCES

[1] Nordberg, E. P. *et al.* Charge sensing in enhancement mode double-top-gated metal-oxide-semiconductor quantum dots. *Applied Physics Letters* **95**, 202102 (2009).
[2] Knapp, T. *et al.* Tuning of two-stage hemt cryogenic amplifier to reduce electron temperature in a nearby quantum dot. *Bulletin of the American Physical Society* **63** (2018).
[3] Davidović, D. *et al.* Tunneling, current gain, and transconductance in silicon-germanium heterojunction bipolar transistors operating at millikelvin temperatures. *Phys. Rev. Applied* **8**, 024015 (2017).
[4] Kalra, R. *et al.* Vibration-induced electrical noise in a cryogen-free dilution refrigerator: Characterization, mitigation, and impact on qubit coherence. *Review of Scientific Instruments* **87**, 073905 (2016).
[5] Beenakker, C. & Schönenberger, C. Quantum shot noise. *Physics Today* **56**, 37–42 (2003).
[6] Kafanov, S. & Delsing, P. Measurement of the shot noise in a single-electron transistor. *Phys. Rev. B* **80**, 155320 (2009).
